# Characterization of the β-barrel assembly machine accessory lipoproteins from *Borrelia burgdorferi*

**DOI:** 10.1186/s12866-015-0411-y

**Published:** 2015-03-24

**Authors:** Joshua P Dunn, Melisha R Kenedy, Henna Iqbal, Darrin R Akins

**Affiliations:** Department of Microbiology and Immunology, University of Oklahoma Health Sciences Center, Oklahoma City, OK 73104 USA

**Keywords:** *Borrelia burgdorferi*, Lyme disease, BAM complex, Lipoproteins, BamA, BamB, BamD

## Abstract

**Background:**

Like all diderm bacteria studied to date, *Borrelia burgdorferi* possesses a β-barrel assembly machine (BAM) complex. The bacterial BAM complexes characterized thus far consist of an essential integral outer membrane protein designated BamA and one or more accessory proteins. The accessory proteins are typically lipid-modified proteins anchored to the inner leaflet of the outer membrane through their lipid moieties. We previously identified and characterized the *B. burgdorferi* BamA protein in detail and more recently identified two lipoproteins encoded by open reading frames *bb0324* and *bb0028* that associate with the borrelial BamA protein. The role(s) of the BAM accessory lipoproteins in *B. burgdorferi* is currently unknown.

**Results:**

Structural modeling of *B. burgdorferi* BB0028 revealed a distinct β-propeller fold similar to the known structure for the *E. coli* BAM accessory lipoprotein BamB*.* Additionally, the structural model for BB0324 was highly similar to the known structure of BamD, which is consistent with the prior finding that BB0324 contains tetratricopeptide repeat regions similar to other BamD orthologs. Consistent with BB0028 and BB0324 being BAM accessory lipoproteins, mutants lacking expression of each protein were found to exhibit altered membrane permeability and enhanced sensitivity to various antimicrobials. Additionally, BB0028 mutants also exhibited significantly impaired *in vitro* growth. Finally, immunoprecipitation experiments revealed that BB0028 and BB0324 each interact specifically and independently with BamA to form the BAM complex in *B. burgdorferi*.

**Conclusions:**

Combined structural studies, functional assays, and co-immunoprecipitation experiments confirmed that BB0028 and BB0324 are the respective BamB and BamD orthologs in *B. burgdorferi*, and are important in membrane integrity and/or outer membrane protein localization. The borrelial BamB and BamD proteins both interact specifically and independently with BamA to form a tripartite BAM complex in *B. burgdorferi*. A working model has been developed to further analyze outer membrane biogenesis and outer membrane protein transport in this pathogenic spirochete.

## Background

The pathogenic spirochetes *Borrelia burgdorferi, B. garinii, and B. afzelii* are the etiologic agents of Lyme disease [1-3]. Although *B. burgdorferi* possesses both a cytoplasmic and outer membrane (OM) similar to Gram-negative bacteria, its OM differs significantly from the typical Gram-negative OM in that it lacks the immunogenic glycolipid lipopolysaccharide [4]. Instead, the borrelial OM contains an abundant number of outer surface lipoproteins, which have been designated Osps [5-23]. The Osps are soluble proteins that are anchored to the bacterial surface by their lipid moiety [6]. In addition to the numerous Osps, the borrelial OM also contains integral outer membrane proteins (OMPs) that contain membrane-spanning domains composed of antiparallel, amphipathic β-strands [23,24]. Presently, only ten OMPs have been identified in *B. burgdorferi* [22,25-33]. However, freeze-fracture electron microscopy studies have revealed that there are numerous OMPs present in the borrelial OM [24]. Therefore, it is likely that the ten known OMPs only represent a small subset and many have likely not yet been identified. Identification and characterization of novel OMPs from *B. burgdorferi* has become an important goal in the field because they may represent novel vaccine candidates.

Exactly how OMPs are folded and inserted into the OM of bacteria is an active area of research [34-43]. While it has been shown that bacterial OMPs (e.g., OmpA, PagP, OmpX, FadL, OmpLA) can spontaneously fold into their native conformation and incorporate into synthetic lipid bilayers and/or detergent micelles *in vitro* [44-47], recent studies have revealed that the lipid composition of bacterial OMs does not readily allow for spontaneous insertion of most OMPs [39]. To overcome this kinetic barrier of insertion *in vivo,* bacteria require the **β**-barrel **a**ssembly **m**achine (BAM) complex [39]. The BAM complex is composed of an essential BamA protein and one or more accessory proteins [34,48-51]. BamA is an OMP itself that contains an N-terminal periplasmic region with five **po**lypeptide **tr**ansport-**a**ssociated (POTRA) domains. POTRA domains are all composed of a highly similar β-α-α-β-β structural motif [35,36] and are important for the interaction between BamA and BAM accessory lipoproteins [52,53]. In *E. coli,* there are four BAM complex accessory lipoproteins: BamB, C, D, and E. BamB and BamD specifically interact with the BamA POTRA domains, while BamC and BamE associate with the larger complex by interacting with BamD [48-50,54,55]. All BAM complexes characterized to date have been shown to be essential for bacterial survival [56-58] due to the critical role they play in OM biogenesis and OMP transport [51,59,60].

While all BAM complexes contain a BamA protein, the accessory lipoproteins can vary greatly in number and overall sequence among different bacterial groups [49,51,53,61-65]. For instance, most β- and γ-proteobacteria typically possess four accessory proteins: BamB, C, D, and E [50,63,66]. In contrast, only BamD orthologs have been identified thus far in the δ- and ε-proteobacteria [49]. We previously reported that the BAM complex in *B. burgdorferi* is comprised of a BamA ortholog and two putative lipoproteins designated BB0324 and BB0028, with BB0324 being suggested to be a BamD ortholog [25,67]. To further these prior analyses, we have used structural studies, functional assays, and co-immunoprecipitation experiments to generate a refined topological model of the *B. burgdorferi* BamA protein and to examine the roles of BB0028 and BB0324 in the borrelial BAM complex. The combined studies revealed that BB0028 and BB0324 play a role in membrane integrity and/or OMP localization, which is consistent with BB0028 and BB0324 being BamB and BamD orthologs, respectively. A working model is proposed for the borrelial BAM complex that can now be used to further explore OM biogenesis and OMP transport in this pathogenic spirochete.

## Results

### Structural model of the *B. burgdorferi* BamA protein

**Figure 1:**
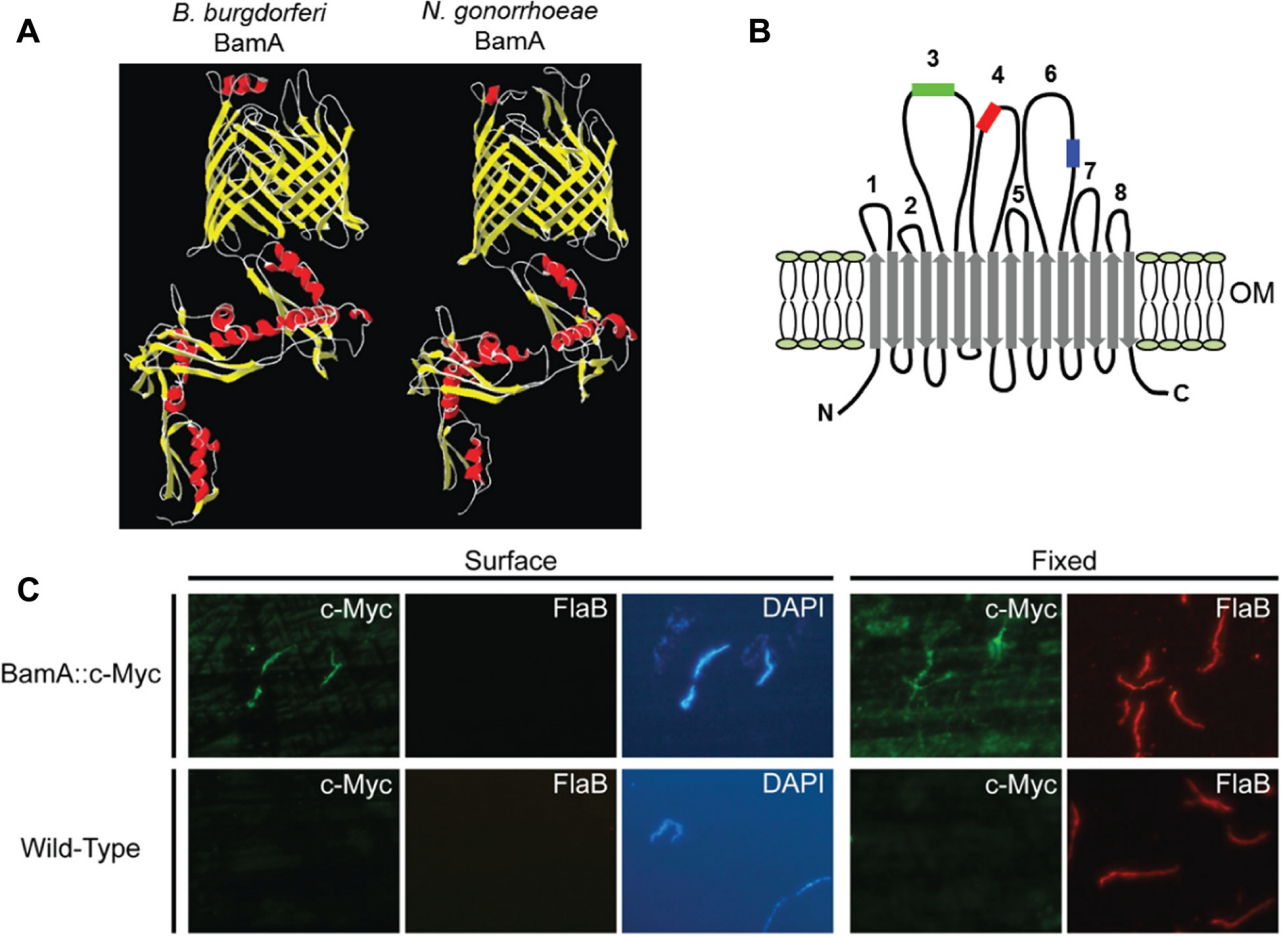
**Proposed structural model of** ***B. burgdorferi*** **BamA. A**. The predicted structure of the *B. burgdorferi* BamA protein (left) determined using the I-TASSER algorithm [68-70] compared to the structure of BamA from *N. gonorrhoeae* (right). Predicted β-sheet regions are depicted in yellow and α-helical regions are depicted in red. **B**. Diagram of the β-barrel domain of the *B. burgdorferi* BamA protein with predicted extracellular loops numbered. The location of the c-Myc tag (EQKLISEEDL) insertion site in extracellular loop three is indicated in green. The site of the predicted α-helical region in extracellular loop four is indicated in red and the blue region within extracellular loop six indicates the position of the conserved RGF motif. OM; outer membrane. **C**. The c-Myc tag inserted in predicted loop three (BamA::c-Myc) was examined using surface immunofluorescence assays and antibodies to c-Myc or the periplasmic FlaB protein (top; two leftmost panels). The DNA-specific dye DAPI was also used to identify all organisms in a given microscopic field (top; third panel from left). As a control for the surface specificity, organisms also were fixed to slides before incubation with the c-Myc and FlaB antibodies (fixed panels). The parental, wildtype strain was also subjected to the same assays (bottom panels).

The *B. burgdorferi* β-barrel assembly machine (BAM) complex appears to be comprised of three proteins: the integral outer membrane protein (OMP) BamA and two accessory lipoproteins designated BB0028 and BamD/BB0324 [25,67]. While there is little information regarding the structure or function of the borrelial accessory lipoproteins, the BamA protein was previously examined using two different structural modeling algorithms to predict the topology of the membrane-spanning C-terminal domain and the conformation of the N-terminal periplasmic region [25]. The recently determined crystal structure of *N. gonorrhoeae* BamA [36], however, provided a known template to refine the structural prediction of the borrelial BamA protein using the I-TASSER protein structure prediction algorithm [68-70]. As shown in Figure 1A, the model predicted for *B. burgdorferi* BamA was observed to be highly similar to *N. gonorrhoeae* BamA, and the confidence score (C-score) for the predicted model was 0.55. The C-score is the most pertinent score for assessing model quality and can range from −5 to +2, with scores > −1.5 considered to accurately indicate the final secondary structure conformation [70]. The I-TASSER server also provides a template modeling score (TM-score) to assess the overall structural similarity between the predicted model and the known template structure (TM-score > 0.5 indicates correct topology prediction). The TM-score for the borrelial BamA model was 0.79 ± 0.09. Combined, the C-score and TM-score indicate that the newly-refined model generated for *B. burgdorferi* BamA is of high quality.

The modeled structure depicted in Figure 1A (left) indicates that *B. burgdorferi* BamA consists of a N-terminal periplasmic domain containing five polypeptide transport-associated (POTRA) motifs followed by a β-barrel domain with 16 membrane-spanning β-strands. A conserved α-helical region was identified in extracellular loop four, which has also been noted in all BamA structures characterized to date [35,36]. Additionally, loop six contains an arginine-glycine-tryptophan triad that may correspond to the RGF motif found in the same loop of other BamA proteins, which has been shown to be essential for BamA function [43,71-73] (Figure 1B). To further examine the reliability of the model generated, we next inserted a c-Myc tag into predicted extracellular loop three (Figure 1B; c-Myc insert shown in green). As shown in Figure 1C, surface immunofluorescence assays using c-Myc-specific antibodies verified that the c-Myc tag, as predicted, was located on the borrelial surface, which is entirely consistent with the structural model predicted by I-TASSER. As expected, the wildtype strain lacking a c-Myc tag displayed no reactivity with the c-Myc antibodies. To ensure that the fragile outer membrane of *B. burgdorferi* was not disrupted, antibodies specific for the periplasmic FlaB protein were also included in the surface localization experiments. When anti-FlaB antibodies were co-incubated with the anti-cMyc antibodies, no FlaB was identified, indicating that the outer membranes were intact (Figure 1C; panels second from left). The DNA-binding dye DAPI was also included in the mounting media for all immunofluorescence experiments to help identify all spirochetes in a given microscopic field. As a control for FlaB reactivity, organisms also were dried to slides and fixed with acetone to disrupt the outer membranes before co-incubation with the FlaB and c-Myc antibodies, as shown in the rightmost panels of Figure 1C. As expected, the fixed organisms fluoresced brightly with the FlaB antibodies.

### Structural models of the *B. burgdorferi* BAM accessory lipoproteins

**Figure 2:**
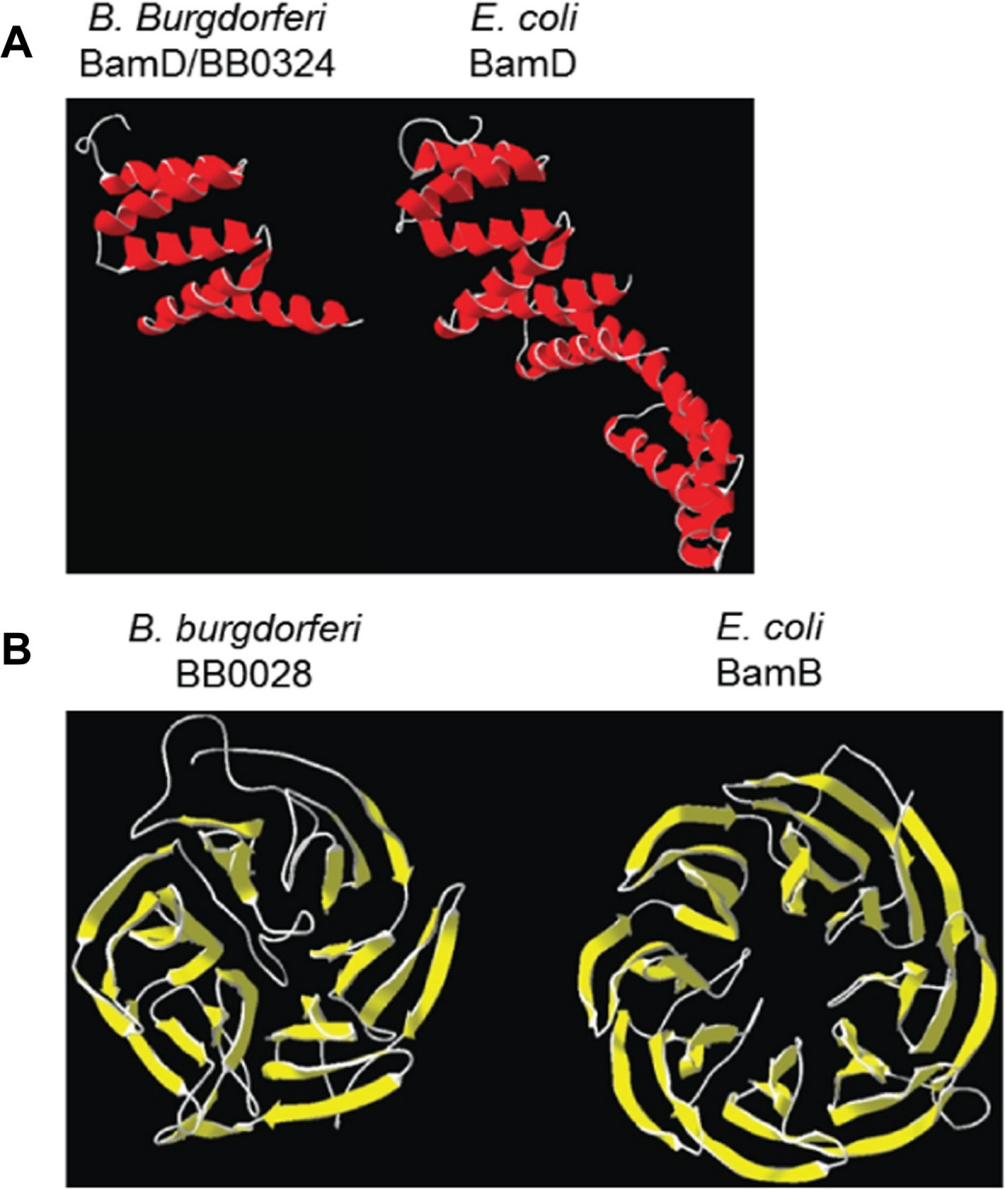
**Structural models of BamD/BB0324 and BB0028.** Structural modeling using the I-TASSER algorithm with predicted β-sheet regions shown in yellow and α-helical regions shown in red. **A**. Predicted structure of *B. burgdorferi* BamD/BB0324 (left) compared to the known structure of the *E. coli* BamD protein (right). **B**. Predicted structure of *B. burgdorferi* BB0028 (left) compared to the known structure of the *E. coli* BamB protein (right).

The *B. burgdorferi* BamA protein has been shown to co-immunoprecipitate with two accessory lipoproteins, encoded by open reading frames *bb0028* and *bb0324* [67]. BB0324 was previously shown to share significant similarity with the N-terminus of BamD from *N. meningitidis* and to contain tetratricopeptide repeat (TPR) domains. TPR domains are antiparallel α-helices commonly involved in mediating protein-protein interactions [74]. The sequence similarity between BB0324 and other known BamD proteins suggested BB0324 is a putative BamD ortholog [67]. BB0028, however, shares no sequence similarity with any of the known or putative BAM accessory proteins. When the I-TASSER algorithm [68-70] was used to model BamD/BB0324 without a specified template, the structure predicted consisted of five stacked α-helical domains (Figure 2A; left). This predicted structure was highly similar to the N-terminal region of the known *E. coli* BamD structure [75,76] (Figure 2A; right). BB0028 was predicted by I-TASSER to fold into a β-propeller structure (Figure 2B; left), which is the known conformation of the *E. coli* BamB protein [52,55,77] (Figure 2B; right).

### Generation of *B. burgdorferi* BamD/BB0324 and BB0028 mutants

**Figure 3:**
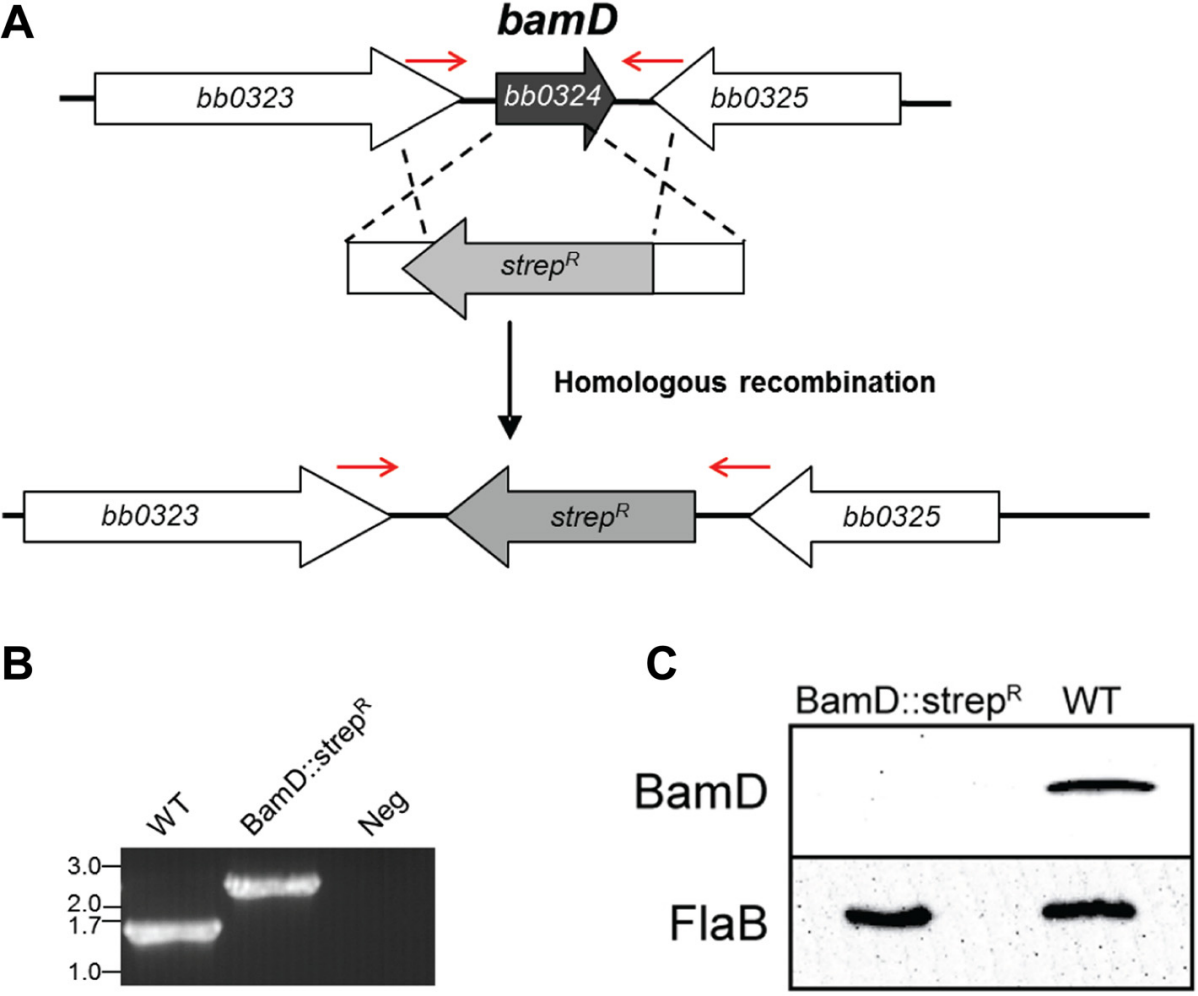
**Generation of a** ***bamD/bb0324*** **mutant. A**. A *bamD*/*bb0324* mutant was generated through homologous recombination by replacing the *bamD/bb0324* gene with a streptomycin resistance cassette. Red arrows indicate primers used to confirm insertion of the streptomycin cassette in the borrelial genome. **B**. PCR amplification resulted in a 1.4 kb amplicon in the wildtype strain while the BamD/BB0324 mutant containing the streptomycin cassette produced a 2.4 kb amplicon, as expected. A PCR reaction with no DNA template (Neg) was included as a negative control. DNA sizes, in kilobase pairs, are indicated at left. **C**. Whole-cell lysate from the BamD/BB0324 mutant (BamD::strep^R^) and parental (WT) strains were subjected to immunoblot analysis using BamD/BB0324 specific antibodies (top panel). FlaB antisera was used to ensure equal loading of lysates examined (bottom panel).

**Figure 4:**
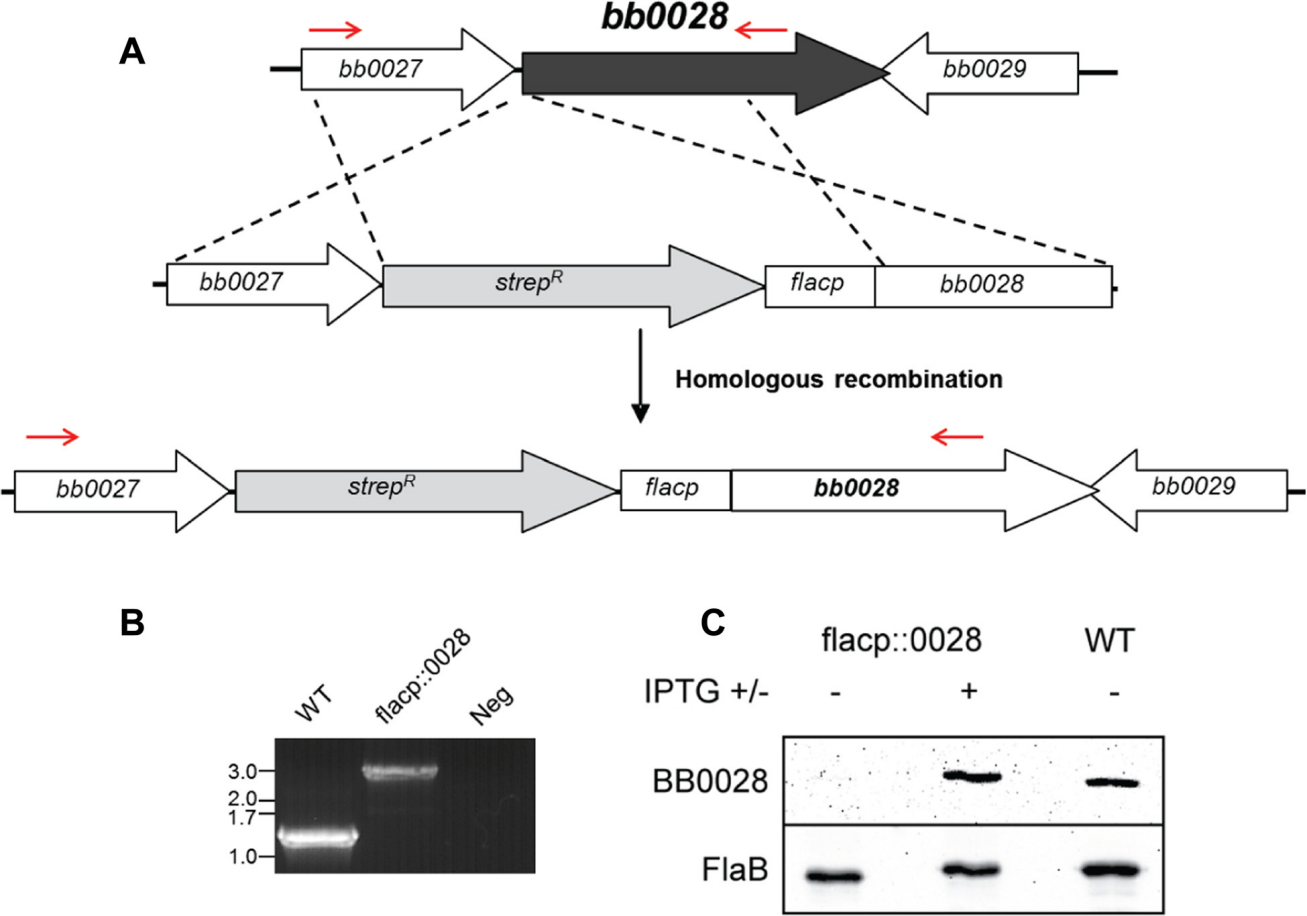
**Generation of a** ***bb0028*** **mutant. A**. An IPTG-regulatable *bb0028* mutant was generated by inserting a streptomycin resistance cassette followed by the *flacp* promoter immediately upstream of *bb0028*. Red arrows indicate primer-binding areas used to confirm insertion of the streptomycin cassette and *flacp* promoter into the borrelial genome. **B**. PCR amplification resulted in a 1.2 kb amplicon in the wildtype strain (WT), as expected, and a 3.0 kb amplicon in the BB0028 mutant (flacp::0028) indicating the streptomycin cassette and *flacp* promoter were inserted in the mutant. A PCR reaction with no DNA template (Neg) was included as a negative control. DNA sizes, in kilobase pairs, are indicated at left. **C**. Whole-cell lysate from the BB0028 mutant (flacp::0028) strain propagated with (+) or without (−) 1 mM IPTG, as well as from the parental, wildtype strain (WT), were subjected to immunoblot analysis using BB0028 specific antibodies (top panel) or FlaB antibodies (bottom panel). FlaB reactivity was used to ensure all lanes were loaded equally.

While prior co-immunoprecipitation experiments [67] combined with the structural modeling outlined above suggest BamD/BB0324 and BB0028 are members of the borrelial BAM complex, neither of these approaches provided empirical evidence that they are functional components of the BAM complex in *B. burgdorferi.* Therefore, to determine if BamD/BB0324 and/or BB0028 function as would be expected of BAM complex accessory proteins, we generated BamD/BB0324 and BB0028 mutants as described in the Methods section. As shown in Figure 3A, *bamD/bb0324* was deleted and replaced by a streptomycin resistance cassette using homologous recombination. The presence of the streptomycin resistance cassette was confirmed by PCR analyses using primers flanking *bamD/bb0324* (Figure 3B). Immunoblot with BamD/BB0324 specific antisera confirmed that the BamD mutant strain, designated BamD::strep^R^, did not express BamD, as expected (Figure 3C). Numerous attempts to delete *bb0028* through homologous recombination were unsuccessful, which suggested BB0028 may be essential for *in vitro* cultivation of *B. burgdorferi*. To overcome this potential caveat, we generated an IPTG-regulatable mutant of BB0028 by expressing the native *bb0028* gene under control of the IPTG-inducible *flacp* promoter (Figure 4A). The resulting strain was confirmed by PCR to contain the streptomycin resistance cassette/*flacp* promoter construct (Figure 4B), and regulation of BB0028 by IPTG was confirmed by immunoblot analysis using polyclonal, monospecific rat anti-BB0028 antibodies (Figure 4C).

**Figure 5:**
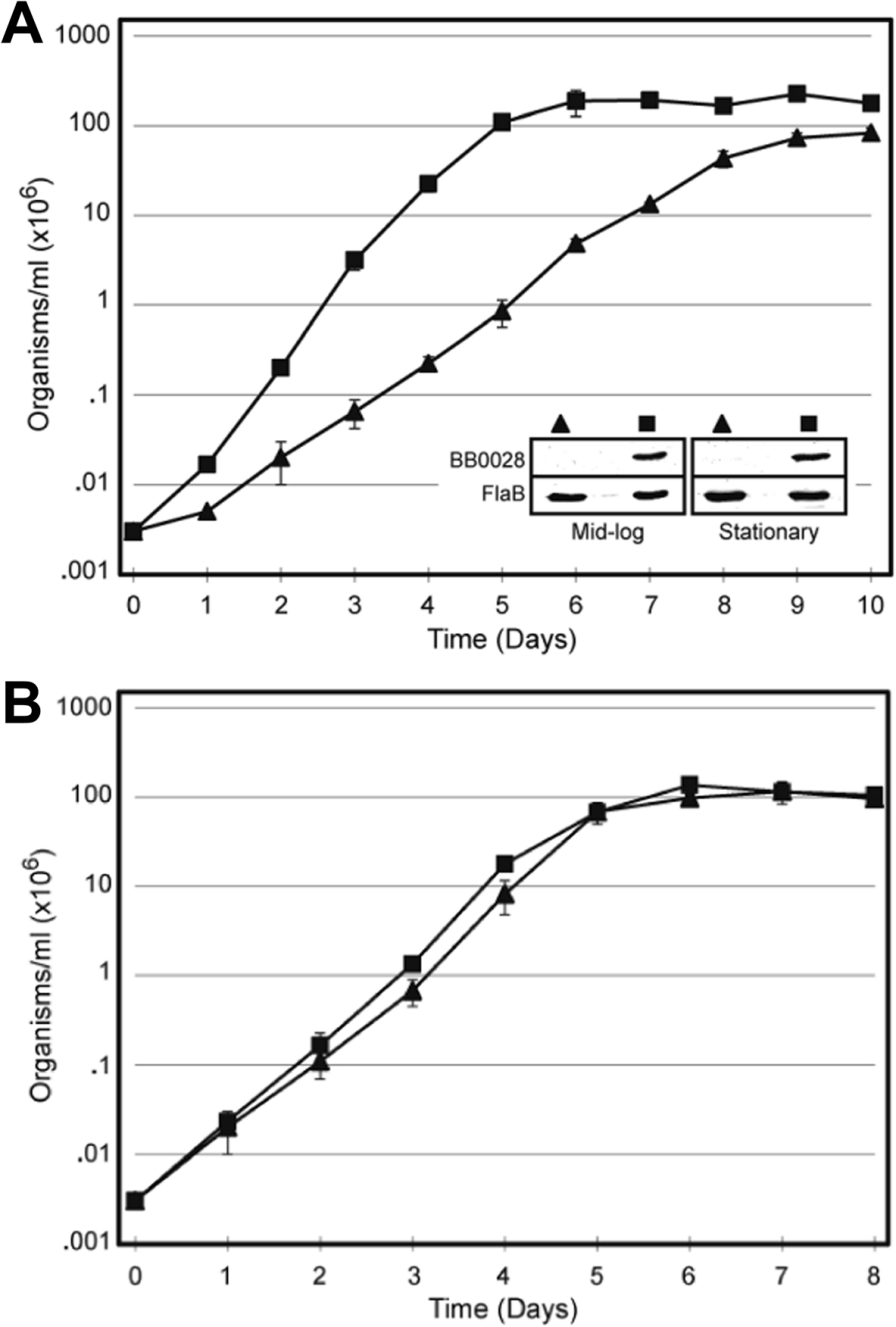
**The BB0028 mutant exhibits impaired growth** ***in vitro.*** **A**. Growth rate analysis of the parental strain (■) and BB0028 mutant strain (▲). The BB0028 mutant required 3–4 more days to reach stationary phase than the parental strain. The inset confirms by immunoblot that BB0028 was not expressed by the BB0028 mutant (▲) during culture. As a control, the wildtype, parental strain (■) was also immunoblotted for BB0028. Lysates also were probed with FlaB specific antibodies to confirm equivalent loading. **B**. Growth rate comparison of the wildtype (■) and BamD/BB0324 mutant (▲) strains.

Interestingly, the BB0028 regulatable mutant was observed to replicate *in vitro* without addition of IPTG in the growth media (Figure 4C), although it should be noted that the organisms were qualitatively less motile when examined under the microscope and replicated at a much slower rate as compared to wildtype organisms expressing normal amounts of BB0028. This observation suggested that BB0028, while not absolutely essential for *in vitro* cultivation of the organism, may be relevant to the overall growth and physiology of *B. burgdorferi*. To more quantitatively examine these observations, we next examined the growth rate of the BB0028 mutant *in vitro*, which revealed that the doubling time of the mutant was 14 h, while the parental strain had a doubling time of only 8 h. The BB0028 mutant also required 72–96 hours longer to reach stationary phase as compared to the wildtype parental strain (Figure 5A). To ensure that the ability of the BB0028 mutant to grow in the absence of IPTG was not the result of a secondary mutation in the *lacI* gene or *flacp* promoter that allowed the mutant to express BB0028, we performed immunoblot analyses which confirmed that BB0028 was not expressed in the mutant strain during cultivation (Figure 5A; inset). When similar growth curve analyses were performed on the BamD/BB0324 mutant strain, no impact on the rate of *in vitro* growth was observed as compared with the parental strain (Figure 5B).

### Loss of BB0028 or BamD/BB0324 enhances antimicrobial sensitivity

In Gram-negative bacteria, mutants lacking various BAM accessory proteins have altered outer membrane (OM) permeability and sensitivity profiles to antimicrobials [64,78,79]. Therefore, we examined whether the absence of the *B. burgdorferi* BAM accessory proteins BB0028 or BamD/BB0324 affected susceptibility to antimicrobial agents. To determine the minimum inhibitory concentration of specific antimicrobials, the respective parental wildtype and mutant strains were cultivated in media containing varying concentrations of carbenicillin, cefotaxime, tetracycline, or minocycline. As shown in Table 1, when compared to the wildtype B31-A3-LK strain, the susceptibility of the BB0028 mutant (flacp::BB0028) to carbenicillin increased by two-fold, and susceptibility to tetracycline increased by four-fold. The BamD/BB0324 mutant (BamD::strep^R^) also exhibited altered susceptibility; it was observed to be two-fold and eight-fold more susceptible to tetracycline and minocycline, respectively, as compared to the parental B31-5A4NP1 strain. We also examined the susceptibility profile of the *B. burgdorferi* IPTG-regulatable BamA mutant [25] as a control. Reducing BamA expression using limiting amounts of IPTG in the regulatable BamA mutant (flacp::BamA) resulted in increased susceptibility to all antibiotics tested, as expected, with a four-fold increase in susceptibility to carbenicillin and two-fold increases to cefotaxime, tetracycline, and minocycline, as compared to the parental strain B31-A3-LK. Taken together, these results suggest that both BB0028 and BamD/BB0324 play important, although possibly different, roles in OM composition and/or integrity.

**Table 1:** **Absence of BamA, BB0028, or BamD/BB0324 affects antimicrobial sensitivity**

	**Minimum inhibitory concentration (ng/ml)** ^***a***^ **(Fold change susceptibility)** ^***b***^			
	**Carbenicillin**	**Cefotaxime**	**Tetracycline**	**Minocycline**
flacp::BB0028^*c*^	156 **(2X)**	20	78 **(4X)**	78
BamD::strep^R^	313	39	39 **(2X)**	10 **(8X)**
flacp::BamA^*d*^	78 **(4X)**	10 **(2X)**	156 **(2X)**	39 **(2X)**
B31-A3-LK	313	20	313	78
B31-5A4NP1	313	39	78	78

*a* – All MIC assays were performed in triplicate.

*b* – flacp::BB0028 and flacp::BamA are compared with strain B31-A3-LK; BamD::strep^R^ is compared with strain B31-5A4NP1.

*c* – flacp::BB0028 was grown in media without added IPTG.

*d* – flacp::BamA was grown in media containing 0.05 mM IPTG.

### Export of BesC into the outer membrane is diminished in the BB0028 mutant

**Figure 6:**
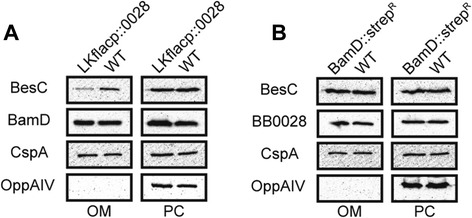
**Absence of BB0028 results in decreased BesC in the** ***B. burgdorferi*** **OM. A**. Outer membrane (OM) and protoplasmic cylinder (PC) fractions were isolated from the BB0028 mutant (LKflacp::0028) and parental (WT) strains for immunoblot analysis using antibodies specific for BesC, BamD/BB0324, CspA, and OppAIV. **B**. OM and PC fractions were isolated from the BamD/BB0324 mutant (BamD::strep^R^) and its parental wildtype (WT) strain and immunoblotted with antibodies specific for BesC, BB0028, CspA, and OppAIV.

To examine the underlying causes for the enhanced susceptibility of the different mutants to antimicrobial agents, we next examined whether the presence of the OMP BesC was altered in the OM of the BB0028 or BamD/BB0324 mutant strains. BesC is the outer membrane protein component of a known *B. burgdorferi* multidrug efflux complex that is involved in enhancing resistance to various antimicrobials [27]. We therefore performed immunoblot analysis on isolated OM fractions to examine the level of BesC exported into the OM of the BB0028 and BamD/BB0324 mutant strains. Interestingly, when equal amounts of the isolated OM fractions were compared, there was a marked decrease in the amount of BesC located in the OM of the BB0028 mutant as compared to its parental wildtype strain (Figure 6A; BesC OM panels). By contrast, no observable decrease in BesC was detected in the OM fraction of the BamD mutant strain as compared to its wildtype parental strain (Figure 6B; BesC OM panels). The overall expression of BesC and its export to the inner membrane also did not appear to be significantly altered in either mutant since BesC levels in the protoplasmic cylinder (PC) fractions were comparable between BB0028 and BamD/BB0324 mutants and their respective parental strains (Figure 6A and B; BesC PC panels). As shown in Figure 6A (BamD panels) and Figure 6B (BB0028 panels), BB0028 and BamD/BB0324 expression levels and OM localization were not affected by the absence of BamD/BB0324 or BB0028, respectively. Membranes were also immunoblotted with antisera to CspA, an OM-localized lipoprotein, to ensure equivalent amounts of PC and OM fractions were loaded for the immunoblot analyses (Figure 6A and B; CspA panels). Finally, OppAIV, a known inner membrane lipoprotein, was used to confirm that the OM fractions were highly purified and devoid of contaminating PC components (Figure 6A and B; OppAIV panels). The combined analyses indicate that BesC export into the OM of *B. burgdorferi* is diminished in the BB0028 mutant but not in the BamD/BB0324 mutant.

### BamA independently interacts with BB0028 and BamD/BB0324

**Figure 7:**
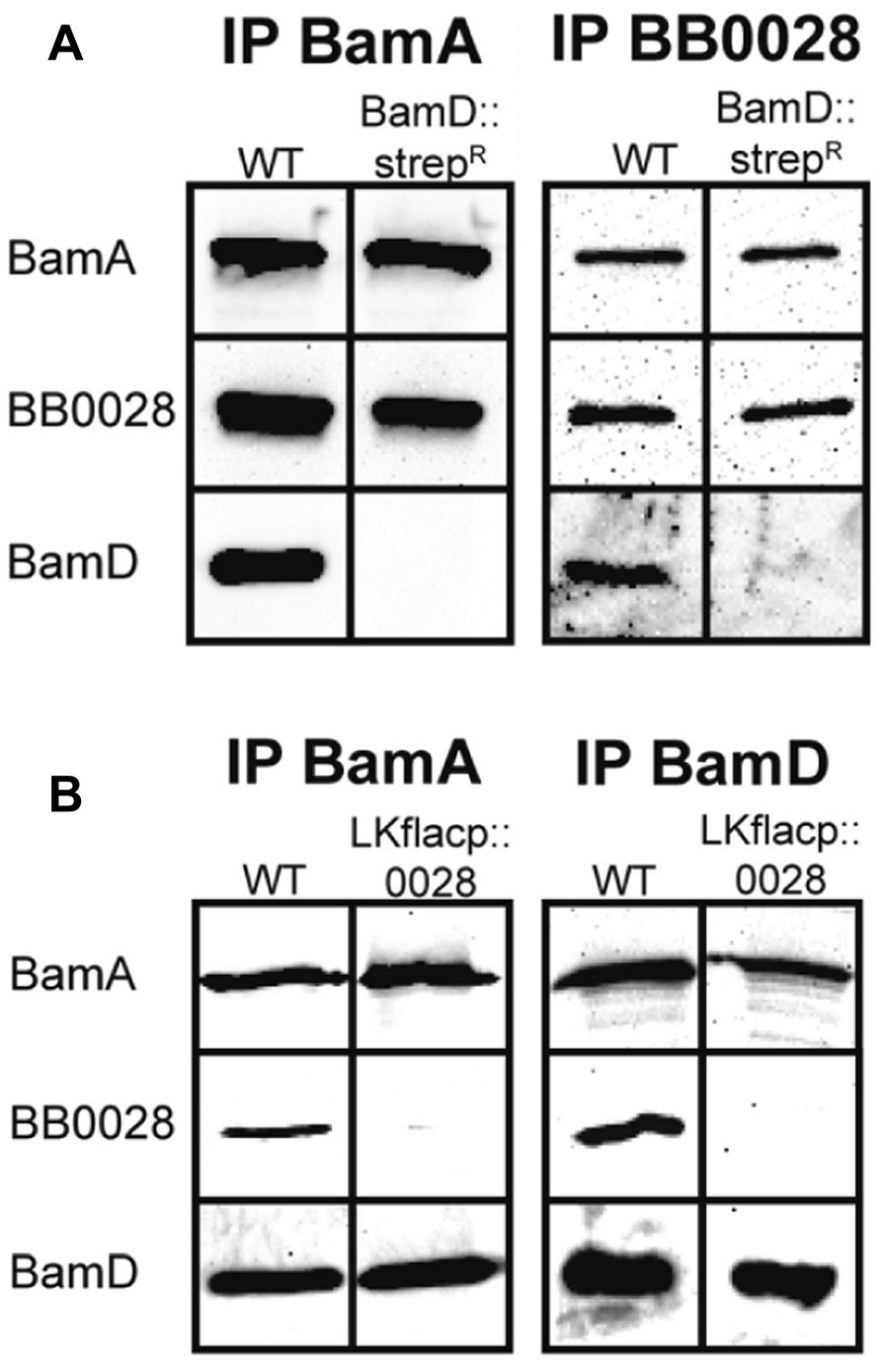
**BB0028 and BamD/BB0324 interact independently with BamA. A**. Co-immunprecipitations of the wildtype strain (WT) or BamD/BB0324 mutant (BamD::strep^R^) using antibodies specific for BamA or BB0028 were performed and subjected to immunoblot with antisera specific to BamA, BB0028, or BamD/BB0324. **B**. Co-immunoprecipitations of the wildtype strain (WT) or BB0028 mutant (LKflacp::0028) strain were performed with antibodies specific for BamA or BamD/BB0324 before immunoblotting with antisera specific to BamA, BB0028, or BamD/BB0324.

We have previously shown that BamA co-immunoprecipitates BamD/BB0324 and BB0028 [67]. These prior studies, however, did not examine whether BamD/BB0324 and BB0028 interact independently with BamA or if they actually interact with each other as a module and only BB0028 or BamD/BB0324 interacts in a specific manner with BamA. Therefore, as shown in Figure 7A, we performed co-immunoprecipitation assays using BamA (left panel) and BB0028 (right panel) specific antibodies in the BamD/BB0324 mutant and wildtype strains. BamA and BB0028 could co-immunoprecipitate each other in the absence of BamD/BB0324. Similarly, as shown in Figure 7B, co-immunoprecipitations with BamA (left panel) and BamD/BB0324 (right panel) specific antibodies revealed that BamA and BamD/BB0324 could co-immunoprecipitate each other in the absence of BB0028. The collective co-immunoprecipitation data indicate that BB0028 and BamD/BB0324 each independently interact with BamA.

## Discussion

### The BAM complex in *B. burgdorferi*

**Figure 8:**
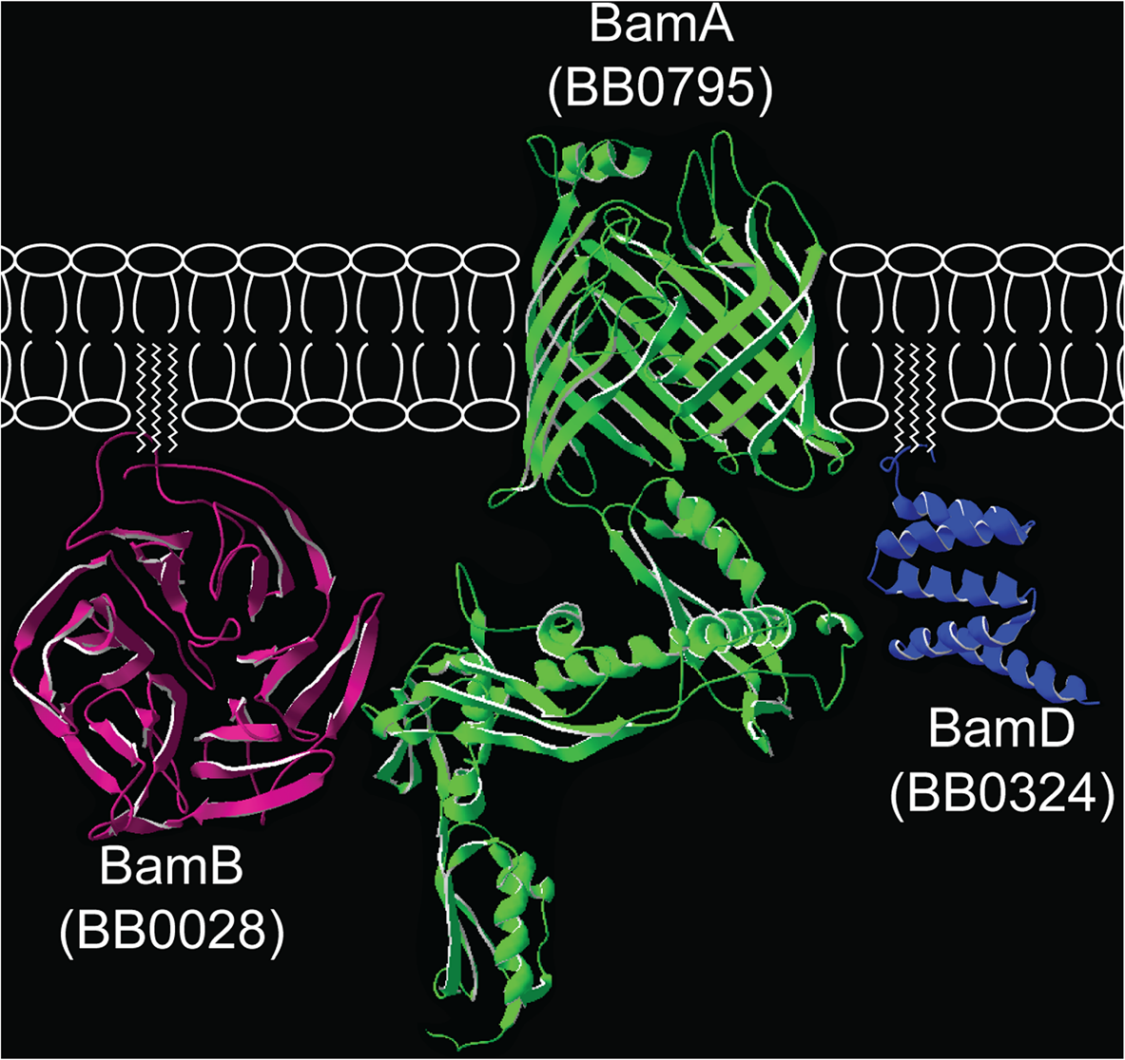
**Current structural model of the tripartite** ***B. burgdorferi*** **BAM complex.** The BAM complex in *B. burgdorferi* deduced from the combined structural models and co-immunoprecipitation data. The accessory lipoproteins BamD (BB0324, blue) and BamB (BB0028, purple) interact independently with the periplasmic POTRA domains of BamA (BB0795, green).

Currently, the predicted composition of the *B. burgdorferi* β-barrel assembly machine (BAM) complex consists of only three proteins: BamA, BB0028, and BamD/BB0324. The combined structural and functional data presented here are consistent with BB0028 and BB0324 being BamB and BamD orthologs, respectively. While we previously suggested that BB0324 is a BamD ortholog [67], the current data fully support the new designation of BamB for BB0028. Hereafter, we will refer to BB0028 as BamB and BB0324 as BamD. Further, the co-immunoprecipitation data revealed that the borrelial BamB and BamD lipoproteins interact specifically and independently with the central BamA protein, which is also entirely consistent with this newly proposed nomenclature. The combined data provided in this report have led us to also propose a new working model for the *B. burgdorferi* BAM complex where BamD and BamB each interact directly and independently with the BamA protein (Figure 8). Future studies will be needed to fully dissect the specific interactions between BamB and BamD with the periplasmic polypeptide transport-associated (POTRA) domains of BamA in *B. burgdorferi*.

### Structural models for the *B. burgdorferi* BamA, BamB, and BamD proteins

The recent crystal structures of BamA from *N. gonorrhoeae*, *H. ducreyi*, and *E. coli* [35,36] allowed us to refine the prior model of the borrelial BamA protein [25]. While all three of these structures are highly conserved, there is only between 49-62% similarity between the *N. gonorrhoeae*, *H. ducreyi*, and *E. coli* BamA proteins. This indicates that while the BamA proteins from different organisms are highly conserved at the three-dimensional level, they are quite divergent at the primary sequence level. For example, the *N. gonorrhoeae* BamA protein shares between 29-52% sequence similarity to BamA orthologs from other bacterial phyla. Accordingly, *B. burgdorferi* BamA is 33% similar to *N. gonorrhoeae* BamA but was predicted to be highly conserved structurally. The model predicted for *B. burgdorferi* BamA contains a C-terminal β-barrel region consisting of 16 transmembrane domains and five POTRA domains along with a unique α-helical region within extracellular loop four, which is a conserved feature that has been identified in all BamA crystal structures reported to date [35,36]. Extracellular loop six in *B. burgdorferi* was predicted to be elongated and associated with the lumen of the protein, which is also similar to the known BamA structures [35,36]. Additionally, as recently highlighted by Albrecht and co-workers [35], extracellular loop six of all known BamA structures contains a conserved three amino acid arginine-glycine-phenylalanine/tyrosine triad, referred to as the RGF motif, which has been shown to be essential for BamA function [43,71-73]. Interestingly, extracellular loop six in the predicted *B. burgdorferi* BamA structure was observed to contain an RGW triad, which is consistent with it corresponding to the RGF motif given that the R and G are identical and the third amino acid is aromatic (W) like in all other RGF motifs identified to date [72]. Future site-directed mutational studies will be required to determine if this RGW triad plays an important role in *B. burgdorferi* BAM function*.*

Structural modeling of the borrelial accessory lipoproteins revealed that the BamB protein folds into a β-propeller, which is a structure conserved among all BamB orthologs [54,77,80]. This structural conservation, combined with its specific interaction with BamA, is consistent with the conclusion that BB0028 is the borrelial BamB ortholog. The *B. burgdorferi* BamD protein was predicted to form a helix-loop-helix structure consisting of five α-helices stacked on top of each other. This same type of folding and helix stacking is also found in the solved structures of the BamD orthologs from *E. coli and Rhodothermus marinus* [49,75,76]. Interestingly, the BamD ortholog in *B. burgdorferi* is C-terminally truncated and is half the size, or less, compared with other BamD orthologs [51,75]. The C-terminal portion of BamD has previously been reported to be integral to mediating the interaction between BamD and BamC in *E. coli* [81]. The apparent lack of a BamC ortholog in *B. burgdorferi* would be consistent with the C-terminal region of BamD being dispensable in *B. burgdorferi*. This finding also further supports the notion that the BAM complex in *B. burgdorferi* is limited to BamA, BamB, and BamD.

### Functional roles of BamB and BamD in *B. burgdorferi*

While we were able to generate a *bamD* null mutant, we were initially unable to generate a *bamB* knockout. To overcome this obstacle, we utilized an IPTG-regulatable promoter system to control expression of BamB so we could examine the role of BamB in the borrelial BAM complex. Interestingly, the BamB regulatable mutant grew in the absence of IPTG and was shown to lack BamB protein expression. This finding suggests BamB is not essential, at least for *in vitro* growth, in *B. burgdorferi*. Lack of BamB expression, however, did impact the overall physiology of the organism as the BamB mutant had a doubling time 75% greater than that of the parental strain. While the BamB null mutant had a marked growth phenotype, which is similar to what has been observed in an *E. coli* BamB mutant [79], it was surprising that the borrelial BamD mutant had no observable growth defect given that BamD orthologs have been reported to be essential in all bacteria studied to date [51,78,81] other than *Salmonella enteritidis* [66]. In the case of *S. enteritidis*, however, while BamD is not essential, the null mutant has a substantially compromised growth rate and reduced viability as compared to the parental strain [66]. Additionally, *S. enteritidis* BamD mutants show reduced amounts of export of OmpA into their OMs [66]. Given that *B. burgdorferi* has at least 10-fold fewer OMPs relative to surface area than the typical Gram-negative organism, this may suggest that the role of BamD is unique in this spirochete [24]. Future studies examining OMP export in the BamD mutant will be required to better understand the role of BamD in the borrelial BAM complex, OMP export, and OM permeability/integrity.

BAM accessory protein mutants have been shown to have altered membrane integrity and/or impaired export of OMPs in other organisms studied [51,64,78,79]. Consistent with this latter observation, we also observed a distinct decrease in the amount of BesC exported into the OM of the BamB mutant, which could explain the differing antibiotic susceptibility profiles observed between the BamB and BamD mutants. The antibiotics we utilized for susceptibility testing typically enter the bacterial cell either through aqueous channels such as porins (i.e., carbenicillin, cefotaxime, and tetracycline) or by traversing the lipid bilayer (i.e., minocycline) [82,83]. Given that BesC can form channels in lipid bilayers [27], it is tempting to speculate that the reduction in the OM of BesC in the BamB mutant correlates with increased susceptibility to the pore-transiting antibiotics carbenicillin and tetracycline. By contrast, the BamD mutant was eight-fold more susceptible to the membrane permeable antibiotic minocycline as compared to the BamB mutant, suggesting that membrane integrity is altered in the BamD mutant, which could lead to enhanced diffusion of this hydrophobic antibiotic across the OM. While future studies will be required to better examine global effects of BamB and BamD on OMP export and membrane integrity, the antibiotic susceptibility and OMP trafficking data presented here provide evidence that both of these BAM accessory proteins play important, although different, roles in *B. burgdorferi* physiology and OM biogenesis.

## Conclusions

In summary, we have shown that *B. burgdorferi* BamA, BamB, and BamD are predicted to be structurally very similar to other known BamA, BamB, and BamD orthologs. Through generation and characterization of BamB and BamD mutants in *B. burgdorferi*, we have shown that BamB has a major effect on physiology and growth rate and both BamB and BamD mutants exhibit altered sensitivity profiles to various antimicrobial compounds. The combined results suggest that they play distinct roles in OMP trafficking and membrane composition. Additionally, the absence of BamB results in a decrease in the presence of the OMP BesC in the borrelial OM, which may account for its altered sensitivity profile to various antibiotics, especially the more hydrophilic antimicrobials such as carbenicillin and tetracycline. BamB and BamD were observed to interact specifically and independently with BamA, which led to our newly proposed working model of the tripartite *B. burgdorferi* BAM complex. The BAM complex model provided here lays the foundation for future studies aimed at dissecting the interaction among the BAM complex components, which should better elucidate OM biogenesis in this unique spirochete.

## Methods

### Bacterial strains and growth conditions

*B. burgdorferi* strains B31cF [84], B31cF BamA::c-Myc, B31-A3-LK [85], B31-5A4NP1 [86], BamD::strep^R^, LKflacp::BamA [25], and LKflacp::0028 were cultivated at 34°C in BSK-II medium containing 6% heat-inactivated rabbit serum (complete BSK-II), supplemented when appropriate with 200 μg/ml kanamycin, 100 μg/ml streptomycin, 40 μg/ml gentamicin, and/or 1 mM or 0.05 mM IPTG. The cloning vector pBluescript-II KS+ (Stratagene, La Jolla, CA) and shuttle vector pBSV2 [87] were propagated using *E. coli* strains DH5α or XL1-Blue (Stratagene) grown in lysogeny broth (LB) or LB agar supplemented with appropriate antibiotics.

### Structural modeling

BamA was modeled by the I-TASSER protein structure and function prediction algorithm [68-70] using the solved crystal structure of *N. gonorrhoeae* BamA [36] as a template. BB0028 and BamD/BB0324 were modeled by the I-TASSER algorithm without selection of a crystal structure template. The resulting structural predictions in .pdb format, as well as the crystal structure .pdb files for *N. gonorrhoeae* BamA [PDB:4K3B], *E. coli* BamB [PDB:2YH3], and *E. coli* BamD [PDB:2YHC], were visualized using Swiss-PdbViewer molecular visualization software [88].

### Generation of antibodies

The DNA sequence encoding BamA POTRA domains P1-P4, plus 14 amino acids of P5, was amplified from *B. burgdorferi* B31 genomic DNA using primers BamA P1 (NheI) F and BamA P4 + 14 (XhoI) R (Primers shown in Table 2). The DNA sequence encoding mature BB0028, lacking the N-terminal leader peptide, was amplified from *B. burgdorferi* B31 genomic DNA using primers *bb0028* (NheI) F and *bb0028* (XhoI) R. The *bamA* P1-4 + 14 and *bb0028* amplicons were then cloned into NheI and XhoI restriction sites of the the pET23a cloning vector (EMD Millipore, Darmstadt, Germany), and the resulting constructs were electroporated into *E. coli* Overexpress™ C41(DE3) (Lucigen Corp, Middleton, WI). His-tagged BamA P1-4 + 14 and BB0028 proteins were then purified as previously described [89,90]. Antibodies were generated against the recombinant BamA P1-4 + 14 and BB0028 proteins by Harlan Bioproducts for Science, Inc. (Madison, WI). Rat antibodies recognizing BamD/BB0324, BesC, CspA, OppAIV, and FlaB were described elsewhere [22,25,67].

**Table 2:** **Oligonucleotides used in this study**

**Name**	**Sequence (5’ to 3’, restriction sites in bold)**	**Description**
BamA P1 (NheI) F	GCG**GCTAGC**AAGGGGAAAATAATAAAGGGTAT	*bamA* nucleotides 82–104 plus NheI site (N-terminus of POTRA 1)
BamA P4 + 14 (XhoI) R	GCG**CTCGAG**ATTTTTATTTTTAGAAACAGTAATAG	Complementary to *bamA* nucleotides 1079–1104 plus XhoI site (14 aa into POTRA 5 domain)
*bb0028* (NheI) F	GCG**GCTAGC**CTGCAAAAAATAAAACATGAATAC	*bb0028* nucleotides 109–132 plus NheI site
*bb0028* (XhoI) R	GCG**CTCGAG**TTCTTTAGTTAATTTTCTGTTTTCC	Complementary to *bb0028* nucleotides 1023–1047 plus XhoI site
795a (BamHI) F	GCG**GGATCC**ATGGGTTCAATTAGAGGTTTGT	*bamA* nucleotides 1–22 plus BamHI site
795a (XbaI) R	GCG**TCTAGA**ATCAGGAACTTCCCTCTTGC	Complementary to *bamA* nucleotides 1562–1581 plus XbaI site
795b (SalI) F	GCG**GTCGAC**CCATTTACAAGTTGGGAAGAAT	*bamA* nucleotides 1582–1593 plus SalI site
795b (PstI) R	GCG**CTGCAG**TCAATATCTCATCTCAATTCCTA	Complementary to *bamA* nucleotides 1562–1581 plus XbaI site
flgB (BamHI) F	GCG**GGATCC**TACCCGAGCTTCAAGGAAGAT	*flgB* promoter nucleotides 1–21 plus BamHI site
flgB (BamHI) R	GCG**GGATCC**ATGGAAACCTCCCTCATTTAAA	Complementary to *flgB* nucleotides 387–408 plus BamHI site
c-Myc (XbaI-SalI) F	GCG**TCTAGA**GAACAAAAACTTATTTCTGAAGAAGATCTG**GTCGAC**GCG	c-Myc tag plus 5’ XbaI and 3’ SalI sites
c-Myc (XbaI-SalI) RC	GCG**GTCGAC**CAGATCTTCTTCAGAAATAAGTTTTTGTTC**TCTAGA**CGC	Complementary to c-Myc tag plus 5’ SalI and 3’ XbaI sites
KO324 downstream (KpnI) F	GCG**GGTACC**GATTATTTGGGCAGATATCAAG	Complementary to nucleotides 330,248-330,269 of B31 chromosome (600 bp downstream of *bb0324*) plus KpnI site
KO324 downstream (XhoI) R	GCG**CTCGAG**TAATTTAAGAAATAAAAATTTTTACTG	Nucleotides 329,622-329,648 of B31 chromosome (immediately downstream of *bb0324*) plus XhoI site
KO324 upstream (BamHI) F	GCG**GGATCC**TTATAGCAATAATAAGCTTATAAAG	Nucleotides 328,661-328,685 of B31 chromosome (600 bp upstream of *bb0324*) plus BamHI site
KO324 upstream (EcoRI) R	GCG**GAATTC**TTCTGCCTCTTTTAGAATGTTTT	Complementary to nucleotides 329,239-329,262 of B31 chromosome (immediately upstream of *bb0324*), plus EcoRI site
flgB (XhoI) F	GCG**CTCGAG**TACCCGAGCTTCAAGGAAG	*flgB* nucleotides 1–21 plus XhoI site
Strep (EcoRI) R	GCG**GAATTC**TTATTTGCCGACTACCTTGGTGAT	Complementary to *aadA* nucleotides 769–792 plus EcoRI site
0028 F 4–29 (EcoRI&NdeI)	GCG**GAATTCCATATG**AAACAAAAATACGAAAACTATTTTAA	*bb0028* nucleotides 4–29 plus EcoRI and NdeI sites
0028 R 678–699 (BamHI)	GCG**GGATCC**ACCACCAGTCATTACTAAAACT	Complementary to *bb0028* nucleotides 678–699 plus BamHI site
0027 F 4–29 (KpnI)	GCG**GGTACC**AGAAAGTATATTTTTATAATACTAAT	*bb0027* nucleotides 4–29 plus KpnI site
0027 R 613–636 (XhoI)	GCG**CTCGAG**CAATTTATTTACATTCACTGTAAC	Complementary to *bb0027* nucleotides 613–636 plus XhoI site

### Generation of *B. burgdorferi* mutants

The c-Myc tag was inserted into a predicted loop of the BamA protein as follows. The first 1,581 nucleotides of *bamA* (open reading frame *bb0795*) were amplified from *B. burgdorferi* B31 genomic DNA using the primers bb0795A (BamHI) F and bb0795A (XbaI) R and were subsequently inserted into the shuttle vector pBSV2 using the restriction sites BamHI and XbaI. The final 860 nucleotides of *bamA* were then PCR-amplified using the primers bb0795B (SalI) F and bb0795 (PstI) R and inserted into the SalI and PstI sites of the vector construct. The borrelial promoter *flgB* was then PCR-amplified from pBSV2 using the primers flgB (BamHI) F and flgB (BamHI) R, and the amplicon was inserted into the vector using the restriction site BamHI. Two 48-mer oligonucleotides, c-Myc (XbaI-SalI) F and c-Myc (XbaI-SalI) RC, encoding the 30-nucleotide c-Myc tag as well as 5’ XbaI and 3’ SalI sites, were then annealed together, digested with XbaI and SalI, and inserted into the construct. Fifteen to 20 μg of the final construct was then electroporated into *B. burgdorferi* strain B31-cF and selected for on the basis of kanamycin resistance to yield strain B31-cF BamA::c-Myc.

The *bamD*/*bb0324* gene was deleted in *B. burgdorferi* strain B31-5A4NP1 by the following method. First, the 600-nucleotide region immediately upstream of *bamD/bb0324* was amplified from *B. burgdorferi* B31 genomic DNA using the primers KO324 upstream (BamHI) F and KO324 upstream (EcoRI) R, and cloned into the pBluescript II KS+ cloning vector. The 600-nucleotide region immediately downstream of *bamD/bb0324* was then amplified with primers KO324 downstream (KpnI) F and KO324 downstream (XhoI) R and inserted into the KpnI and XhoI sites of the vector. A cassette containing the *flgB* promoter and *aadA* streptomycin resistance gene was then amplified from the pKFSS1 [91] plasmid using the primers flgB (XhoI) F and Strep (EcoRI) R, and the amplicon was inserted into the vector using XhoI and EcoRI. The final construct was then electroporated into *B. burgdorferi* strain B31-5A4NP1 and selected with 100 μg/ml streptomycin to yield strain BamD::strep^R^. Potential positive clones were screened by PCR using the primers KO324 upstream (BamHI) F and KO324 downstream (KpnI) F, and immunoblotted with anti-BamD/BB0324 antibodies to verify the absence of BamD/BB0324 in whole cell lysates. The final BamD::strep^R^ isolate was subjected to complete plasmid analysis and was found to possess all plasmids except cp9, which was previously shown to be absent from the parental B31-5A4NP1 strain [86].

To generate a regulatable BB0028 mutant, the hybrid *flacp* promoter [85] was inserted upstream of the *bb0028* gene. Nucleotides 4–699 of *bb0028* were amplified from *B. burgdorferi* B31 genomic DNA using the primers 0028 F 4–29 (EcoRI&NdeI) and 0028 R 678–699 (BamHI) and cloned into the multiple cloning site of the pBluescript II SK+ cloning vector using the restriction sites EcoRI and BamHI. A cassette containing the constitutive *flgB* promoter, the *aadA* streptomycin-resistance gene, and the hybrid *flacp* promoter was then digested from the pTLflacp::795 construct [25] previously generated in our laboratory and cloned into the XhoI and NdeI sites of the vector. Finally, nucleotides 4–636 of *bb0027* were amplified using primers 0027 F 4–29 (KpnI) and 0027 R 613–636 (XhoI), and the resulting amplicon was then inserted upstream of the streptomycin-resistance cassette using the restriction enzymes KpnI and XhoI. The final construct was subsequently electroporated into *B. burgdorferi* strain B31-A3-LK and grown in the presence of 1 mM IPTG and selected with streptomycin to yield strain LKflacp::0028. Potential positive clones were screened by PCR using the primers 0027 F 4–29 (KpnI) and 0028 R 678–699 (BamHI). To verify IPTG-regulation of BB0028 from the *flacp* promoter, whole-cell lysates were immunoblotted with anti-BB0028 antibodies. The final LKflacp::0028 isolate was subjected to a complete plasmid analysis and was found to possess all plasmids except cp9, which was previously shown to be absent from the parental B31-A3-LK strain [85,92,93].

### Surface localization assay

*B. burgdorferi* strain B31-cF BamA::c-Myc and wildtype B31-cF organisms were enumerated and diluted to a concentration of 5 × 10^6^ cells per ml in complete BSK-II medium. For surface localization assays, wildtype B31-cF and BamA::c-Myc-expressing cell suspensions were then co-incubated with rabbit anti-c-Myc antibody (Sigma-Aldrich, St. Louis, MO, dilution of 1:20) and rat anti-FlaB (dilution of 1:500) for one hour. The cells were gently pelleted at 4000 × *g* and washed three times with PBS. The final pellet was resuspended in 100 μl PBS, and 10 μl aliquots were spotted on glass slides and allowed to air dry overnight. Slides were subsequently fixed for 10 minutes with acetone and then blocked for 30 minutes in PBS containing 0.5% BSA. Samples were incubated for 45 minutes with Alexa-Fluor 488-conjugated goat anti-rabbit antibodies (1:250 dilution; Life Technologies, Grand Island, NY) and Alexa-Fluor 568-conjugated goat anti-rat antibodies (1:500 dilution; Life Technologies). After incubation, all samples were washed three times in blocking buffer and mounted in buffered glycerol containing 4’,6-diamidino-2-phenylindole dihydrochloride (DAPI; Vector Laboratories, Burlingame, CA). As a control, samples were also washed, spotted onto glass slides, and fixed with acetone prior to incubation with rabbit anti-c-Myc antibody and rat anti-FlaB for one hour. Slides were visualized at 1000X magnification on an Olympus BX60 fluorescent microscope (Olympus America Inc, Center Valley, PA).

### Immunoblotting

After blocking in milk buffer composed of phosphate buffered saline, 5% nonfat dried milk, and 0.5% Tween, polyvinylidine fluoride membranes were incubated for one hour with rat antisera at the following dilutions in milk buffer: 1:2000 for anti-BamA, anti-BB0028, anti-OppAIV, and anti-BesC; 1:1000 for anti-BamD and anti-CspA. Membranes were then washed three times, incubated in milk buffer with a 1:10,000 dilution of HRP-conjugated goat anti-rat antibodies (Bio-Rad, Hercules, CA), and washed three additional times. Immunoblots were developed by enhanced chemiluminescence (Amersham Biosciences, Piscataway, NJ).

### Growth assays

Triplicate 14 ml cultures were seeded at 3000 *Borrelia*/ml and grown at 34°C in complete BSK-II containing appropriate antibiotics and IPTG as necessary. Cultures were then enumerated at 24-hour intervals by dark field microscopy until reaching stationary phase, and the mean *Borrelia*/ml were plotted for each strain at each time point. To ensure appropriate IPTG regulation of BB0028 at all phases of growth, samples of the LK and LKflacp::0028 strains were taken at mid-log (approx. 1 × 10^7^*Borrelia*/ml; day 4 for LK, day 6 for LKflacp::0028) and stationary (day 6 for LK, day 10 for LKflacp::0028) phases, and whole-cell lysates were prepared and immunoblotted with rat BB0028 antibodies. Standard deviation for each timepoint was determined and is shown by error bars in Figure 5.

### Antimicrobial susceptibility assays

Antimicrobial susceptibility was determined by a method adapted from Bunikis, et al. and Hunfeld, et al. [27,94]. Antimicrobial agents were serially diluted in a 96-well microtiter plate in a volume of 100 μl complete BSK-II per well. Each well was then inoculated with 100 μl of complete BSK-II media containing strain-specific antibiotics and IPTG, if necessary, and 5 × 10^7^*Borrelia*/ml, for a final cell density of 2.5 × 10^7^*Borrelia*/ml. The concentration ranges for each antimicrobial agent tested were as follows (ng/ml): carbenicillin 2–5000; cefotaxime 5–10,000; tetracycline 5–10,000; and minocycline 5–10,000. Plates were then sealed with laboratory film and incubated for 72 hours at 34°C with 5% CO_2_. At 0-, 24-, 48-, and 72-hour timepoints, the absorbance of each well was measured at 562 nm and 630 nm using a Molecular Devices SpectraMax 340 plate reader (Molecular Devices, Sunnyvale, CA). Each assay was repeated in triplicate, and the mean 562/630 nm ratio, corresponding to the amount of growth in each well, for each concentration of each antimicrobial agent was then plotted with respect to time to determine the minimum inhibitory concentration of each antibiotic.

### Isolation of *B. burgdorferi* OM vesicles

*B. burgdorferi* outer membrane (OM) and protoplasmic cylinder (PC) fractions were isolated as previously described [25,95,96]. Equivalent amounts of OM and PC fractions were then separated by SDS-PAGE and immunoblotted with appropriate antibodies. To ensure equivalent loading of OM and PC fractions, membranes were immunoblotted for the OM lipoprotein CspA [22,97]. To ensure proper fractionation of OM and PC fractions, membranes were also immunoblotted for the lipoprotein OppAIV, which is known to localize to the inner membrane [96,98].

### Co-immunoprecipitation

Lysates of *B. burgdorferi* strains B31-5A4NP1, BamD::strep^R^, B31-A3-LK, and LKflacp::0028 were prepared for co-immunoprecipitation studies as previously described [67]. Co-immunoprecipitation was performed according to manufacturer’s protocol using the Pierce Crosslink Immunoprecipitation Kit (Pierce Biotechnologies, Rockford, IL). Briefly, 250 μl lysate from each strain was pre-cleared and then applied in IP/Lysis Buffer to Protein A/G columns treated and crosslinked with 10 μl of antiserum to BamA, BB0028, or BamD. After incubation at 4°C for 3 hours, columns were washed and the bound protein was eluted in low pH elution buffer for later analysis by SDS-PAGE and immunoblotting.

## Abbreviations

BAM: β-barrel assembly machine
OM: Outer membrane
OMP: Outer membrane protein
POTRA: Polypeptide transport-associated
TPR: Tetratricopeptide repeat
PC: Protoplasmic cylinder
